# Infertility, IL-17, IL-33 and Microbiome Cross-Talk: The Extended ARIA-MeDALL Hypothesis

**DOI:** 10.3390/ijms252211981

**Published:** 2024-11-07

**Authors:** Samir Hamamah, Fatima Barry, Sarah Vannier, Tal Anahory, Tari Haahtela, Josep M. Antó, Charles Chapron, Jean-Marc Ayoubi, Wienczyslawa Czarlewski, Jean Bousquet

**Affiliations:** 1 Biologie de la Reproduction, Hôpital Arnaud de Villeneuve, 34295 Montpellier, France; fatima.barry@chu-montpellier.fr (F.B.); t-anahory@chu-montpellier.fr (T.A.); 2 INSERM DEFE, Université de Montpellier, 34090 Montpellier, France; 3 Gynécologie Médicale, Hôpital Arnaud de Villeneuve, 34295 Montpellier, France; s-vannier@chu-montpellier.fr; 4 Skin and Allergy Hospital, Helsinki University Hospital, 00250 Helsinki, Finland; tari.haahtela@haahtela.fi; 5 ISGlobal, Barcelona Institute for Global Health, 08036 Barcelona, Spain; josepm.anto@isglobal.org; 6 IMIM (Hospital del Mar Medical Research Institute), 08003 Barcelona, Spain; 7 Departamento de Ciencias Experimentales y de la Salud, Universitat Pompeu Fabra (UPF), 08002 Barcelona, Spain; 8 CIBER Epidemiología y Salud Pública (CIBERESP), 28029 Madrid, Spain; 9 Service de Gynécologie-Obs., Hôpital Cochin, 75014 Paris, France; charles.chapron@gmail.com; 10 Gynécologie et médecine de la Reproduction, Hôpital Foch, 92150 Suresnes, France; jm.ayoubi@hopital-foch.com; 11 MASK-air, 34000 Montpellier, France; 12 Charité-Universitätsmedizin Berlin, Corporate Member of Freie Universität Berlin and Humboldt-Universität zu Berlin, 10117 Berlin, Germany; jean.bousquet@orange.fr; 13 Fraunhofer Institute for Translational Medicine and Pharmacology ITMP, Allergology and Immunology, 12203 Berlin, Germany

**Keywords:** infertility, endometriosis, IL-17, IL-33, estrogens, dysbiosis

## Abstract

Infertility, defined as the inability to obtain pregnancy after 12 months of regular unprotected sexual intercourse, has increased in prevalence over the past decades, similarly to chronic, allergic, autoimmune, or neurodegenerative diseases. A recent ARIA-MeDALL hypothesis has proposed that all these diseases are linked to dysbiosis and to some cytokines such as interleukin 17 (IL-17) and interleukin 33 (IL-33). Our paper suggests that endometriosis, a leading cause of infertility, is linked to endometrial dysbiosis and two key cytokines, IL-17 and IL-33, which interact with intestinal dysbiosis. Intestinal dysbiosis contributes to elevated estrogen levels, a primary factor in endometriosis. Estrogens strongly activate IL-17 and IL-33, supporting the existence of a gut–endometrial axis as a significant contributor to infertility.

## 1. Introduction

Infertility is defined as the inability to conceive after 12 or more months of regular, unprotected sexual intercourse. In recent decades, the prevalence of infertility has risen, becoming a major public health problem. Assisted reproductive technologies (ART) are considered the most efficient tool. However, success rates have only marginally increased over the past decades, despite significant and constant advances in ART [1]. Various lifestyle risk factors have been linked to infertility, with nutrition being one of the key factors [2]. Noninfectious infertility is often associated with endometriosis or polycystic ovary syndrome (PCOS). Endometriosis is an estrogen-dependent chronic inflammatory process, whereas PCOS is associated with androgens. Endometriosis is a chronic gynecological condition in which tissue similar to the endometrium, the lining of the uterus, grows outside the uterine cavity, commonly affecting the ovaries, fallopian tubes, and pelvic peritoneum. This ectopic endometrial tissue responds to hormonal changes during the menstrual cycle, often leading to inflammation, scarring, and adhesions. Endometriosis is a significant cause of chronic pelvic pain, dysmenorrhea, and infertility [3]. The prevalence of endometriosis is estimated to affect approximately 10% of reproductive-age women worldwide, although this figure may be higher due to underdiagnosis [4]. The condition can have a substantial impact on quality of life and poses a major challenge in reproductive health.

Polycystic ovary syndrome (PCOS) is a complex endocrine disorder characterized by hormonal imbalance, metabolic abnormalities, and reproductive dysfunction. It is defined by the presence of at least two of the following criteria: irregular or absent menstrual cycles (oligo- or anovulation), hyperandrogenism (excess levels of androgens causing symptoms like hirsutism, acne, and hair thinning), and polycystic ovaries observed on ultrasound. The exact cause of PCOS remains unclear, but it is believed to involve a combination of genetic, hormonal, and environmental factors that lead to insulin resistance and ovarian dysfunction. PCOS is a leading cause of infertility due to its impact on ovulation and is also associated with increased risks of metabolic syndrome, type 2 diabetes, cardiovascular disease, and mood disorders. PCOS affects an estimated 5% to 20% of women of reproductive age worldwide, depending on the diagnostic criteria used, with prevalence rates generally around 10% in most populations [5]. The disorder has significant implications for women’s health, not only impacting reproductive outcomes but also contributing to long-term health risks. Early diagnosis and management are essential to address both the reproductive and metabolic aspects of the condition.

Interleukin (IL) IL-17 is a cytokine secreted by T helper 17 (Th17) cells, a subset of CD4+ T cells that differentiate in the presence of cytokines like TGF-β and IL-6. These Th17 cells are key in immune responses against bacterial and fungal infections. Other cells, such as γδ T cells, natural killer (NK) cells, and neutrophils, can also produce IL-17, especially during infections and inflammatory conditions. Its expression is primarily restricted to barrier surface tissues such as the intestine, gingiva, conjunctiva, vaginal mucosa, and skin. IL-17 is produced in low amounts in response to the beneficial resident microbiota, stimulating the production of antimicrobial peptides by epithelial cells to maintain a balanced bacterial and fungal population. There is a delicate balance between IL-17 and the microbiota; dysbiosis is known to lead to Th17 suractivation and elevated IL-17 production in an attempt to restore equilibrium. Disruption of the healthy microbiota contributes to the development of various chronic inflammatory and autoimmune diseases, partly by affecting Th17 cell responses in the gut, which, in turn, influences systemic Th17 activation. Additionally, several cytokines, influenced by lifestyle factors, regulate IL-17 differentiation and its persistence in tissues during chronic inflammation. Excessive IL-17 activity is associated with chronic inflammation and has been implicated in autoimmune diseases and reproductive disorders, as it promotes the recruitment of neutrophils and enhances local inflammation in affected tissues. In immunologicdiseases, such as psoriasis or rheumatoid arthritis, IL-17 blockage has been very effective [6,7].

IL-33 is part of the IL-1 cytokine family and serves as a warning signal for the immune system in response to epithelial or endothelial cell damage caused by necrosis, infection, allergies, stress, or trauma. It is mainly released by nonimmune cells, including epithelial and endothelial cells, in response to cellular stress or damage [8]. It is also produced by various immune cells, such as mast cells, macrophages, and dendritic cells, and interacts with its receptor ST2, which is expressed on the surface of Th2 cells, regulatory T cells, and innate lymphoid cells (ILCs). IL-33 plays a significant role in type 2 immune responses and has been linked to the development of several conditions, including allergic, cardiovascular, autoimmune, neurodegenerative, infectious diseases, and cancer. Interestingly, IL-33 can either contribute to disease improvement or exacerbate its progression. Genetic variations in the IL33 gene may influence whether an individual is more likely to be immune or susceptible to certain diseases [9].

## 2. The ARIA-MeDALL Hypothesis

The ARIA-MeDALL hypothesis refers to a model that integrates insights from the “Allergic Rhinitis and its Impact on Asthma” (ARIA) initiative and the “Mechanisms of the Development of Allergy” (MeDALL) project. This hypothesis proposes that allergic diseases, particularly in childhood, do not occur in isolation but are interconnected and often follow a pattern known as the “allergic march”, where different allergic conditions (such as eczema, food allergies, asthma, and allergic rhinitis) develop and overlap over time. The ARIA-MeDALL hypothesis suggests that genetic, environmental, and immunological factors interact to drive a unified mechanism underlying the onset and progression of multiple allergic diseases.

**Figure 1:**
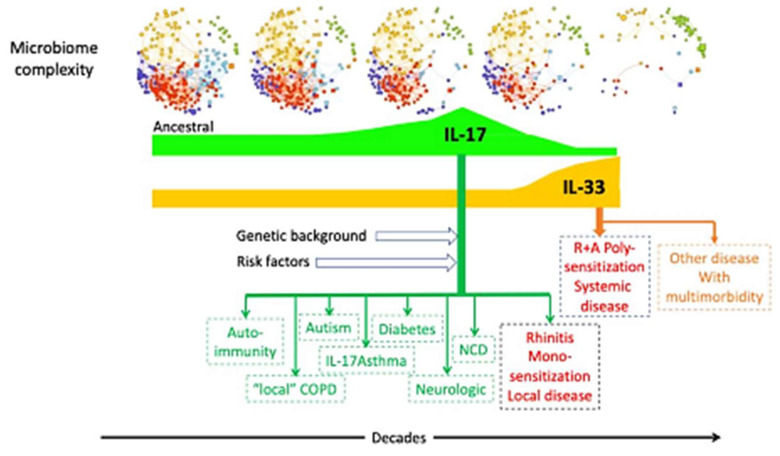
Putative mechanisms between microbiome and diseases.

Recent findings have led to the formulation of the ARIA-MeDALL hypothesis that rhinitis alone may be a distinct entity from rhinitis and asthma multimorbidity (A + R) with clinical and therapeutic relevance. This hypothesis is supported by several factors: (i) distinct gene expression profiles observed in rhinitis, which show a preference for toll-like receptor and IL-17 expression, as opposed to A + R, where IL-33 and IL-5 are more prominently expressed [10]; (ii) differing allergen sensitization patterns, with rhinitis typically involving mono- or pauci-sensitization, while A + R tends to show polysensitization [11,12]; (iii) the greater symptom severity observed in epidemiological studies [13,14]; (iv) real-life studies; and (v) differences in treatment response. The hypothesis focuses on the roles of IL-17 and IL-33 and their interactions with the microbiome, along with various co-factors. Depending on genetic factors (such as toll-like receptors (TLR) and IL-33 variants), environmental influences, and other defined or unknown elements, the interaction between these cytokines and the microbiome varies. In the context of a healthy ancestral microbiome, IL-17 performs its typical protective role. However, as the microbiome’s diversity diminishes, IL-17 can become pathogenic, engaging with TLRs to drive localized disease. In cases of rhinitis, there is IgE production in response to a limited range of allergens. With further loss of microbiome complexity, the IL-33 pathway becomes activated, leading to multimorbidity (asthma and rhinitis) and polysensitization in individuals who are genetically predisposed (Table 1, Figure 1). 

## 3. The Female Reproductive Tract Microbiota and Infertility

The following terms will be used according to Jiang [15] (Table 2).

The composition of the vaginal microbiota plays a crucial role in maintaining the integrity of the cervical epithelium and supporting the protective functions of the cervical barrier against infections. Microbial communities vary depending on the areas of the reproductive tract [16]. *Lactobacillus* species represent the main microorganisms of vaginal and endometrial microbiota [17]. Like gut microbiota, vaginal microbiota can be modified by many factors, including hormones and westernized lifestyle [18,19]. 

### 3.1. The Female Reproductive Tract (FRT) Microbiota and Endometriosis

Potential explanations for the role of dysbiosis in endometriosis include the Bacterial Contamination Theory, immune system activation, cytokine-induced disruption of gut function, altered estrogen metabolism and signaling pathways, and imbalances in progenitor and stem cell homeostasis [15]. 

Women with endometriosis face a significantly higher risk of lower genital tract infections. Sampling of FRT microbiota has been effective in predicting both the risk and stage of the disease [15]. Endometriotic microbiotas are associated with diminished *Lactobacillus* dominance and increased elevated abundance of vaginosis-related bacteria and other opportunistic pathogens [15]. Women with endometriosis exhibit higher levels of colony-forming units of *Gardnerella*, *Streptococcus*, *Enterococci*, and *Escherichia coli* in the endometrium. In the cervix, while *Atopobium* is absent, there is an increase in *Gardnerella*, *Streptococcus*, *Escherichia*, *Shigella*, and *Ureaplasma* [20]. Variety in the cervical microbiome has been linked to improved clinical outcomes in endometriosis [21]. The cervical microbiome may shift during the development and progression of the disease, with increased *Firmicutes* and decreased *Actinobacteria* and *Bacteroidetes*. Unlike the vaginal microbiome, the upregulation of *Lactobacillus*, along with higher levels of *Streptococcus* and lower levels of *Dialister*, is frequently associated with advanced stages of endometriosis, severe pain, and infertility. A notable reduction in the richness and diversity of the cervical microbiome has been significantly observed in patients with more severe clinical symptoms [21].

Vaginal dysbiosis (microbial imbalance associated with adverse effects on host health) can lead to vaginal infections (such as mycoses or bacterial vaginosis) [22]. Vaginal dysbiosis was associated with infertility [16]. 

There is a fine balance between the pathogenic and protective functions of IL-17 in the female reproductive tract. IL-17 plays an important role in maintaining the homeostasis of the microbiome and the immune response generated during fungal, bacterial, and viral infections associated with protection but also with inflammation. The relevance of IL-17 has been demonstrated in bacterial, fungal, and viral infections within the female reproductive tract, where the innate and adaptive production of IL-17 is involved in a variety of immunomodulatory processes, including neutrophil recruitment, DC regulation, and Th1 modulation [22]. IL-17 expression is upregulated in serum, peritoneal fluid (PF), and endometriotic lesions from patients with endometriosis, especially in the early stages of the disease [23,24]. IL-17 is involved in endometriosis in the regulation of immune microenvironment, and the invasion and growth of ectopic lesions [23]. IL-17 may be associated with pelvic pain [25] (Table 3).

Dysbiosis-induced IL-33 contributes to impaired antiviral immunity in the genital mucosa [34]. In endometrium, IL-33 perpetuated inflammation, angiogenesis, and lesion proliferation, which are critical events in the progression of endometriosis [45,46,47]. In a 2013 prospective study involving 151 patients, Santulli et al. [48] demonstrated for the first time that elevated serum levels of IL-33—a cytokine known for its role in fibrotic disorders—are associated with the presence of uterine leiomyoma.

### 3.2. The FRT Microbiome and PCOS

The vaginal microbiome is also involved in PCOS with a reduction in *Lactobacillus* sp. [49,50]. Vaginal bacterial species among PCOS patients may be associated with testosterone levels [51]. Nonetheless, it remains unclear whether the vaginal microbiome influences the onset or progression of PCOS [52]. Few studies have shown that IL-17 and IL-33 were involved in PCOS [53,54,55].

### 3.3. Treatment of Endometriosis or PCOS by Alteration of the Genital Microbiome

Although research is still in its early stages, there is growing evidence that antibiotic and probiotic treatments may offer therapeutic benefits for managing endometriosis [15]. Further research, including randomized controlled trials, is needed to establish standardized treatment protocols and fully understand how these therapies can be integrated into the management of endometriosis. Nonetheless, these approaches offer a potential avenue for novel, microbiome-targeted therapies that could improve patient outcomes.

## 4. The Gut Microbiome and Infertility

The gut microbiome plays a critical role in regulating various aspects of physiology, metabolism, and immune function, with its activity closely linked to nutritional intake. The abundance and metabolic activity of gut microbiota follow a diurnal circadian rhythm, primarily controlled by host nutrition and hormones [56]. This delicate balance between the gut microbiome, immune responses, and physiological states has been disrupted by westernized diets [57]. However, the composition of the gut microbiome differs significantly from that of the genital microbiome [58].

In cases of endometriosis, altered gut microbiota profiles have been observed, and these changes appear to contribute to the progression of the disease, suggesting a bidirectional interaction [59]. Additionally, these microbiomes may influence the gut–brain axis, potentially explaining the link between endometriosis and symptoms such as infertility and chronic pelvic pain [60]. The gut microbiota is also modulated by estrogens and, in turn, affects estrogen levels [61]. An increased presence of β-glucuronidase-producing bacteria can lead to higher circulating estrogen levels, which may promote the development and progression of endometriosis [61].

PCOS, a common cause of infertility, is also involved in gut microbiome changes [52,62]. In PCOS, dysbiosis with an imbalance of particular bacterial species such as *Bacteroidetes* and *Firmicutes* [63] was associated with an altered production of short-chain fatty acids and suggested a role for IL-22 and bile [61,63]. The changes in microbiome in PCOS are similar to those of metabolic dysregulation [49]. Androgens appear to regulate the gut microbiome in females and androgen excess may be linked with gut dysbiosis in PCOS. Women with PCOS have an excess of androgens in relation to estrogen. The altered gut microbiota in PCOS may promote increased androgen biosynthesis and decreased estrogen levels through lowered beta-glucuronidase activity. PCOS is frequently linked to systemic metabolic issues, including hyperinsulinemia, insulin resistance (IR), obesity, and a heightened risk of type II diabetes and cardiovascular diseases [64]. Thus, it is possible that multimorbid PCOS may be associated with changes in microbiome. 

## 5. Estrogens, Dysbiosis, IL-17, and IL-33

Three primary factors that disrupt estrogen availability in women with endometriosis are the expression of estrogen-synthesis enzymes, the estrobolome, and the metabolome [15]. The activity of the estrobolome regulates the amount of excess estrogen that is either eliminated from or reabsorbed into the body [65]. When this activity is disrupted, often due to imbalances in the gut microbiome, excess estrogen may be retained in the body. This estrogen can then enter the bloodstream and be transported to the endometrial and peritoneal tissues [65]. This process leads to a hyperestrogenic state, which fuels the progression of endometriosis, offering a potential explanation for how gut microbiota dysbiosis might contribute to the disease. Additionally, the metabolome, which is significantly shaped by gut microbiota activity, plays a key role in the gut–brain axis [66]. Gut metabolites stimulate the production of estrogens. Imbalances in the gut microbiota, or dysbiosis, can lead to alterations in the metabolome, resulting in elevated levels of serotonin, glutamate, short-chain fatty acids (SCFAs), and gamma-aminobutyric acid (GABA). These metabolites can reach the brain, where they stimulate the production of estrogens [15]. 

**Figure 2:**
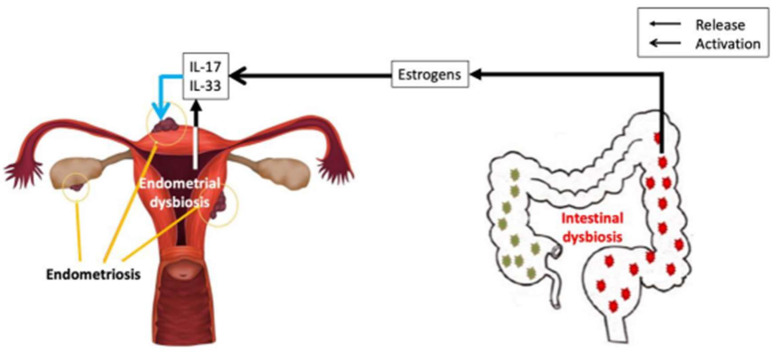
Cross-talk between intestinal and endometrial dysbiosis in endometriosis.

Many different mechanisms can explain gut dysbiosis and endometriosis but can also propose a role for IL-17 and IL-33 (Figure 2). Estrogen signaling enhances the release of IL-33 in response to allergens, promotes cytokine production by ILC2 cells, and amplifies airway inflammation [67]. IL-33 is involved in allergic and nonallergic inflammation [8]. IL-33-group 2 innate lymphoid cells are regulated by estrogens in the uterus [68]. Estrogens enhance IL-17 expression in uterine γδ T cells [69]. The important activation of IL-17 and IL-33 pathways by estrogens provides a link between uterine and gut microbiomes. Interestingly, the recent study of Wang et al. (Table 3) found that, in women with chronic endometritis (CE) and recurrent implantation failure (RIF), there is a significant dysregulation in cytokine expression and autophagy markers. Specifically, the expression of IL-17 was markedly higher in these patients compared to controls, while the levels of IL-10 and TGF-β were significantly lower [29]. Furthermore, autophagy marker LC3-II expression was elevated, and the mTORC1 pathway was impaired in CE patients. These findings suggest that CE is associated with a shift toward a proinflammatory environment in the endometrium, driven by an imbalance favoring Th17 over Treg responses, and exacerbated by altered autophagy processes. This dysregulated immune environment is likely to contribute to reduced endometrial receptivity and increased risk of implantation failure.

## 6. Male Infertility, Cytokines, and Microbiome

IL-17 has been linked to male infertility, as it negatively impacts both sperm motility and viability [33,70]. This cytokine promotes inflammatory responses that can lead to oxidative stress and tissue damage in the male reproductive system. Such inflammation can impair the functionality of sperm by disrupting the environment in which they develop and function, leading to reduced motility and compromised sperm viability [71]. Additionally, the presence of IL-17 in the reproductive tract may contribute to the breakdown of sperm membrane integrity, further diminishing their capacity to fertilize an egg. This association suggests that increased IL-17 levels could play a detrimental role in male reproductive health by creating a hostile environment for sperm survival. Sabbaghi et al. [72] showed that IL-17A levels were elevated in both the seminal plasma and blood serum of varicocele patients compared to the control group. Additionally, this cytokine level was higher in the follicular fluid of patients with endometriosis, polycystic ovary syndrome (PCOS), and tubal factor infertility than in the control group.

The composition of the seminal microbiome can significantly influence male fertility. Emerging research suggests that the presence and balance of specific microorganisms in semen may affect sperm health, motility, and overall reproductive potential. An imbalance or dysbiosis in the seminal microbiome, characterized by the overgrowth of pathogenic bacteria or the reduction in beneficial microbes, has been linked to conditions such as reduced sperm quality, oxidative stress, and inflammation of the male reproductive tract, all of which can impair fertility. Furthermore, the seminal microbiome may also play a role in influencing the immune environment within the reproductive system, potentially impacting successful fertilization. Understanding the interactions between microbial populations in semen and male reproductive health could lead to new insights and treatments for male infertility [73]. A diverse semen microbiome was identified with modest similarity to the urinary microbiome. Infertile men harbored increased seminal α-diversity and distinct β-diversity [74]. In a meta-analysis [75], it was found that *Ureaplasma urealyticum*, *Enterococcus faecalis*, *Mycoplasma hominis*, and *Prevotella* negatively impact semen parameters, whereas *Lactobacillus* appears to protect sperm quality. 

A clear link has been established between gut microbiota dysbiosis and male infertility. Disruptions in the balance of gut bacteria can lead to systemic effects that influence reproductive health. Specifically, an imbalanced gut microbiome can contribute to increased inflammation, oxidative stress, and metabolic dysfunctions, such as insulin resistance and obesity, which are known factors that negatively impact sperm quality and overall male fertility. Additionally, the gut microbiota plays a crucial role in regulating hormonal balance, including the metabolism of testosterone and other androgens essential for reproductive function. Alterations in gut bacteria can therefore indirectly affect spermatogenesis and impair sperm motility, morphology, and concentration [74,76,77,78]. 

Recent research has begun to uncover the complex interplay between immune factors, particularly cytokines IL-17 and IL-33, the microbiome, and semen quality, offering new insights into male infertility. IL-17 and IL-33, known for their roles in inflammatory and immune responses, appear to influence reproductive health by affecting the inflammatory environment within the male reproductive system. Elevated IL-17 levels, typically associated with chronic inflammation, have been linked to increased oxidative stress in the testes and seminal fluid. This oxidative stress can damage sperm DNA, impair motility, and decrease sperm viability, all of which are critical parameters of semen quality [79]. Similarly, IL-33, which is involved in both inflammation and tissue repair, may exacerbate inflammation when dysregulated, contributing to a hostile environment in the testes and epididymis. Excessive IL-33 activation can lead to immune cell infiltration, further elevating oxidative stress and impairing the structural integrity of sperm [80].

These cytokine effects are further compounded by dysbiosis in the male genital and gut microbiome. The microbiome plays a crucial role in maintaining immune balance, as microbial communities regulate inflammatory responses and modulate cytokine levels. An imbalanced microbiome, particularly gut dysbiosis, can result in elevated systemic inflammation, leading to heightened levels of IL-17 and IL-33. Dysbiosis within the male genital tract itself can disrupt the protective microbial environment necessary for optimal sperm health, contributing to oxidative stress and inflammatory damage [81]. Studies have shown that certain microbial communities in the gut can influence hormone regulation and immune signaling, suggesting a gut–testis axis through which dysbiosis may indirectly impair semen quality by modulating IL-17 and IL-33 levels [82]. This novel connection implies that treatments targeting microbial balance, such as probiotics, alongside anti-inflammatory interventions, may offer potential therapeutic benefits for improving semen quality and, consequently, male fertility. Integrating this understanding of cytokine and microbiome interactions may reshape approaches to diagnosing and managing male infertility, focusing on reducing inflammation and restoring microbial health to optimize semen parameters [83].

## 7. Relationship Between Infertile Male and Female Microbiotas 

Unprotected sexual intercourse facilitates the exchange of bacteria between partners, allowing each to influence the microbiota composition within the other’s reproductive tract [84]. Investigating bacterial transmission through sexual activity presents challenges, as fluctuations in the female microbiota are influenced by multiple factors beyond the microbial contribution from semen. These factors include hormonal changes, variations associated with the menstrual cycle, and broader environmental or lifestyle influences. The complexity of these interactions makes it difficult to isolate the specific effects of bacterial exchange during intercourse on the female reproductive microbiome, as it is constantly shaped by both internal and external variables [85]. Considering how female microbiota may act upon male microbial fauna [56], it may also be tempting to hypothesize that a couple living in the same environment have the same dietary patterns and that a similar dysbiosis may exist in couples with male and female infertility, leading to increased infertility.

## 8. Discussion

The links between infertility and the microbiome are complex. They involve many different mechanisms and various sites of the microbiome (genital or gut). However, some hypotheses can be proposed.

Endometriosis and PCOS risks have genetically, developmentally, and physiologically opposite causes [86]. They show opposite patterns of prevalence within populations: where one is more common, the other is less common. This is supported by data of variation among populations in levels of prenatal testosterone, which mediate risks of both conditions [86]. A low level of prenatal testosterone favors the occurrence of endometriosis [87]. Testosterone downregulates IL-17 [88,89]. Testosterone levels are decreased when microbiome diversity is reduced [90]. Given that hormones like estrogen and testosterone have a substantial impact on cytokine pathways (including IL-17 and IL-33), accounting for hormonal fluctuations across the menstrual cycle or during reproductive treatments should be an integral part of study designs. Since lifestyle factors like diet, stress, and pollutant exposure can affect the microbiome, it is important to control for these variables—such as by using questionnaires or tracking diet. This approach helps to isolate the direct effects of cytokines on infertility, minimizing potential biases from these external influences.

The ARIA-MeDALL hypothesis in infertility is based on the reduction in gut and uterine microbiome diversity by westernized lifestyle. Endometriosis, an estrogen-associated disease, is a heterogeneous disease. Some forms may be associated with gut and uterine dysbiosis, with a gut–endometrium axis promoting endometrial IL-17 and IL-33 pathways enhanced by estrogens. On the other hand, PCOS may be an androgen-associated disease with an impact on the gut (and possibly vaginal) microbiome, although some estrogen pathways can also be activated. This hypothesis may explain, at least partly, some of the dysbiosis impact on fertility due to westernization of the microbiome.

## 9. Conclusions

The connections between infertility and the microbiome are multifaceted, involving diverse mechanisms and multiple microbiome sites, such as the gut and genital tract. Alterations in the microbiome can impact hormone regulation, immune responses, and inflammatory pathways, all of which play key roles in reproductive health. For instance, dysbiosis in the gut microbiome may disrupt estrogen metabolism, influencing levels of circulating estrogens that are critical for fertility. In the genital tract, an imbalanced microbiome can lead to inflammation and immune dysregulation, which may impair implantation and pregnancy maintenance. The ARIA-MeDALL hypothesis, originally linking chronic diseases to dysbiosis and proinflammatory cytokines like IL-17 and IL-33, has recently been extended to infertility, proposing that dysbiosis-driven inflammation and hormonal disruptions may similarly contribute to reproductive challenges. While these mechanisms are still being explored, the emerging “gut-reproductive axis” and “genital microbiome health” hypotheses suggest that restoring microbial balance could be a potential strategy for improving fertility outcomes.

## Figures and Tables

**Table 1:** The ARIA-MeDALL hypothesis.

In Allergic and Airway Diseases
The hypothesis focuses on IL-17, IL-33, and their interactions with the microbiome and co-factors. The relationship between these cytokines and the microbiome varies depending on genetic background (such as TLR, IL-33, and other genes), environmental exposures, and other defined or undefined factors.
In a microbiome with ancestral complexity, IL-17 performs its normal protective function. However, as microbiome diversity diminishes, IL-17’s role shifts to a pathogenic one, interacting with TLRs (local disease) and other mechanisms. For instance, in cases of rhinitis, there is IgE production to a limited number of allergens, and it is probable that co-factors like viral infections contribute to disease onset. This disease commonly appears after childhood.
With a further reduction in microbiome diversity, the IL-33 pathway becomes activated. In genetically susceptible individuals, this leads to multimorbidity and polysensitization. Such activation can occur shortly after birth (atopic march) or later in early childhood, associated with factors like viruses, *Staphylococcus aureus*, pollutants, or nonallergenic components of allergens.
IL-33 may also downregulate IL-17 pathways.
In other noncommunicable diseases and autoimmune conditions, this hypothesis similarly centers on the roles of IL-17, IL-33 (or other pivotal cytokines), and their interactions with the microbiome

**Table 2:** Glossary of terms used (from Jiang [15]).

Term	Definition
Microbiota	The community of microorganisms, including bacteria, archaea, protists, fungi, and viruses, that live in and on the human body.
Microbiome	The complete set of genetic material belonging to the microbiota.
Estrobolome	The collection of genes within the gut microbiota that are involved in estrogen metabolism.
Metabolome	The full set of metabolites found in a particular environment.
Dysbiosis	A disruption or imbalance in the microbiota, marked by the presence of harmful microbes or a reduction in beneficial ones.
Prebiotic	Substances that stimulate the growth and activity of beneficial microorganisms.
Probiotic	Live microorganisms that provide health benefits to the host.

**Table 3:** IL-17 and IL-33 in fertility.

Author	Year	Study Group	Main Results
**IL-17 mouse**			
Anipindi [26]	2016	Vaginal cells of mice treated by estradiol or placebo	Estradiol Boosts CD4+ T-Cell Antiviral Immunity by Preparing Vaginal Dendritic Cells to Trigger Th17 Responses through an IL-1-Dependent Mechanism.
**IL-17 human**			
Rajaei [27]	2011	Case control, N = 12 + 10	Undetectable IL-17
Crosby [28]	2020	20 infertile women	Increase in the IL-17A pathway in endometrial tissue from women with unexplained infertility affects pregnancy outcome following assisted reproductive treatment.
Wang [29]	2019	Case–control, N = 75 endometriosis + 75 normal	Increased expression of IL-17 and decreased IL-10-TGFß in endometriosis possibly associated with Treg (mTORC1 autophagy)
Zhao [30]	2021	47 infertile women after blastocyst transfer	Same profile the day of transplantation. IL-17 increase at day 3 in all women. But persistence of increased IL-17 at days 6 and 9 only in women with negative transplantation.
Olkowska- Truchanowicz [31]	2021	Case–control: 36 endometriosis and 26 none	Peritoneal fluid IL-17 increased in endometriosis stages I–II by comparison to no-endometriosis and endometriosis stages III–IV. IL-17 more increased when endometriosis was associated with infertility. IL-1ß and VEGF increased in endometriosis.
Wu [24]	2021	36 endometriosis	IL-17 and MAPK signaling pathway significantly enriched in the eutopic endometria of women with endometriosis
Jiang [32]	2022	48 women with stage III–IV endometriosis	Interleukin-17 Receptor E (IL-17RE) and CD27 were the labels of heterogeneous peritoneal fluid Th17 subsets
Mary [33]	2021	cross-sectional study, 39 men with infertility diagnosed based on semen analysis and 39 subjects with normal semen analysis	In infertile cases, MMP-9 and IL-17 were significantly increased when compared with controls (*p* = 0.046 and *p* = 0.041, respectively). A significant association of MMP-9 was observed with IL-17 (*p* = 0.037)
**IL-33 mouse/rat**			
Oh [34]	2016	mice	Dysbiosis-induced IL- 33 contributes to impaired antiviral immunity in the genital mucosa
Begum [35]	2020	mice	Dynamic Expression of Interleukin-33 and ST2 in the Mouse Reproductive Tract Is Influenced by Superovulation
Valero-Pacheco [36]	2022	mice	Maternal IL-33 critically regulates tissue remodeling and type 2 immune responses in the uterus during early pregnancy in mice
Kozai [37]	2021	rats	Protective role of IL33 signaling in negative pregnancy outcomes associated with lipopolysaccharide exposure
Wu [38]	2015	mice	IL-33 is shown to be associated with follicle atresia and required for disposal of degenerative tissue during ovarian atresia
**IL-33 human**			
He [39]	2022	Case–control, N = 20 + 20	Reduced intracellular IL-33 levels negatively affect endometrial receptivity in women with adenomyosis. This is linked to a correlation with HOXA10 expression. IL-33 enhances endometrial receptivity by promoting STAT3 phosphorylation
Bourdon [40]	2019	Case–control N = 60 + 20	Low IL-33 (and IL-17F) in adenomyosis group
Lin [41]	2021	Cells from endometrioma	IL-33 promotes invasiveness of human ovarian endometriotic stromal cells through the ST2/MAPK/MMP-9 pathway activated by 17b-estradiol
Southcombe [42]	2013	N = 21 + 64	Soluble ST2 detected in Human Follicular Fluid and Luteinized Granulosa Cells. sST2 levels increased in the largest follicules.
Kaitu’u-Lino [43]	2012	Case–control, N = 150 + 20 abortions	Increase in maternal serum IL-33 and soluble ST2 associated with miscarriage. It is proposed that an increase in IL-33 may be a T2 compensatory response to prevent abortion.
Moretti [44]	2021	44 human semen samples/control group of 11 fertile men	IL-33 was absent in all analyzed semen samples, suggesting a role of a nuclear factor rather than secreted cytokine in human seminal plasma

## Data Availability

No new data were created or analyzed in this study. Data sharing is not applicable to this article.
